# Monitoring of amyloid related imaging abnormalities: SWI vs T2*-GRE

**DOI:** 10.1016/j.tjpad.2025.100220

**Published:** 2025-06-18

**Authors:** Diana M. Sima, Thanh Vân Phan, Ana M. Franceschi, Wende N. Gibbs, Frederik Barkhof, Philip Scheltens, Stephen Salloway, Jeffrey Cummings, Wim Van Hecke, Dirk Smeets

**Affiliations:** a icometrix, Leuven, Belgium; b Department of Radiology, Donald and Barbara Zucker School of Medicine at Hofstra/Northwell, Lenox Hill Hospital, New York, NY, USA; c Department of Radiology, Barrow Neurological Institute, Phoenix, AZ, USA; d Queen Square Multiple Sclerosis Centre, Department of Neuroinflammation, UCL Queen Square Institute of Neurology, Faculty of Brain Sciences, University College London, London, UK; e Centre for Medical Image Computing (CMIC), Department of Medical Physics and Biomedical Engineering, University College London, London, UK; f Department of Radiology and Nuclear Medicine, Amsterdam UMC location VUmc, Amsterdam, Netherlands; g Institute for Health Research (NIHR), University College London Hospitals (UCLH) Biomedical Research Centre (BRC), London, UK; h Alzheimer Center Amsterdam, Department of Neurology, Vrije Universiteit Amsterdam, Amsterdam UMC location VUmc, Amsterdam, the Netherlands; i Amsterdam Neuroscience, Neurodegeneration, Amsterdam, the Netherlands; j EQT Life Sciences, Amsterdam, the Netherlands; k Butler Hospital, Memory and Aging Program, and Warren Alpert Medical School of Brown University, Providence, RI, USA; l Department of Brain Health, Chambers-Grundy Center for Transformative Neuroscience, Kirk Kerkorian School of Medicine, University of Nevada Las Vegas (UNLV), Las Vegas, NV, USA

**Keywords:** Amyloid-related imaging abnormalities (hemorrhagic) (ARIA(-H)), Cerebral microbleeds (CMB), Superficial siderosis, T2*-gradient recalled echo (T2*-GRE), Susceptibility weighted imaging (SWI)

## Abstract

Amyloid-β–directed monoclonal antibody therapies may lead to amyloid-related imaging abnormalities (ARIA). Clinical trials that formed the basis for the ARIA radiographic severity grading scale adopted by the approved drugs’ labels utilized T2* gradient recalled echo (T2*-GRE) images for ARIA-hemorrhagic (ARIA-H) assessment. Little is known about the application of susceptibility-weighted imaging (SWI) to ARIA-H assessment. We exploited comparative studies on the usage of SWI instead of 2D T2*-GRE and simulated the impact of SWI’s higher sensitivity on the derived ARIA-H severity distribution for three approved drugs. The simulations indicated that the two sequences are not equivalent when grading ARIA-H severity and that the rate of therapy discontinuation would increase by more than 50% compared to the rates reported in the drugs’ prescribing information. This should be taken into consideration whenever SWI is applied for ARIA safety monitoring. Appropriate imaging guidelines are needed to enhance management of amyloid-β-directed antibody therapies.

## Dear editor

Amyloid-β–directed monoclonal antibody therapies have shown promise in slowing Alzheimer’s disease. An increasing number of patients are now on these treatment protocols. However, the treatments have potentially significant complications that can lead to morbidity or mortality, and require protocol modification. Therefore, monitoring their side-effects with brain MRI is crucial for patient safety. One of the side-effects is hemosiderin deposition, typically appearing as cerebral microbleeds (CMB) or superficial siderosis (SS), also called amyloid-related imaging abnormalities - hemorrhagic (ARIA-H) microhemorrhages and ARIA-H superficial siderosis, respectively.

Clinical trials that formed the basis for the ARIA radiographic severity grading scale [1] adopted by the approved drugs’ labels utilized T2*-gradient recalled echo (T2*-GRE) images for ARIA-H assessment. Therapeutic decision-making is based on this severity: treatment is suspended or discontinued for moderate-or-severe ARIA-H microhemorrhages, i.e., when the count of new CMB is above or equal to 5, or moderate-or-severe ARIA-H superficial siderosis, i.e., when the count of SS is above or equal to 2.

Meanwhile, susceptibility-weighting imaging (SWI) became the standard in many clinical practices. This is motivated by the fact that SWI offers an increased sensitivity compared to T2*-GRE. However, little is known about its application to ARIA-H assessment.

To estimate the impact of different acquisition sequences (T2*-GRE, SWI with thick or thin slice) on the derived radiographic severity for ARIA-H [1], we performed a Monte Carlo simulation, where we initialized the probability distributions of CMB and SS so that they matched the prevalence observed in the clinical trials of aducanumab, lecanemab and donanemab. The initial distribution of CMB was then modulated based on findings of Shams et al. (2015, Tables 1-2) [2] on a cohort of 246 subjects with AD, mild cognitive impairment and subjective cognitive impairment, assessed with 3 MRI sequences (T2*-GRE, thick-slice SWI and thin-slice SWI) by 4 raters. Averaged over the raters, the CMB detection rate was 1.5 times higher for thick-slice SWI versus T2*-GRE, and 1.9 times higher for thin-slice SWI versus T2*-GRE. Similarly, the initial distribution of SS was modulated based on findings of Assis Lopes (2024, Table 2) [3], where shifts in SS severity evaluations and higher multifocality scores for thin-slice SWI compared to T2*-GRE were observed on a cohort of 54 patients with cerebral amyloid angiopathy assessed by 2 raters. Averaged over the raters, the rate of multifocal SS detection was 1.3 times higher for thin-slice SWI versus T2*-GRE.

Our Monte Carlo simulation (*N* = 100,000 samples) resulted in an ARIA-H microhemorrhages severity distribution that matched well the observed distributions in the respective clinical trials for the T2*-GRE approach, but led to an increased prevalence and severity for SWI (Table 1). The estimated proportion of patients with moderate-or-severe ARIA-H microhemorrhages (whose therapy would be suspended or discontinued) would increase from 5.6 % (aducanumab), 4.6 % (lecanemab), 8.8 % (donanemab) when employing T2*-GRE, to 7.8 % (aducanumab), 6.2 % (lecanemab), 11.7 % (donanemab) when employing thick-slice SWI, and to 8.9 % (aducanumab), 7.0 % (lecanemab), 13.5 % (donanemab) when employing thin-slice SWI. The latter corresponds to a relative increase of more than 50 % (61 % for aducanumab, 54 % for lecanemab and 53 % for donanemab), compared to T2*-GRE (which was used to establish the drug labels). Furthermore, multifocal SS (i.e., moderate-or-severe ARIA-H superficial siderosis) would increase from 7.5 % (aducanumab), 1.3 % (lecanemab), 9.1 % (donanemab) when employing T2*-GRE, to 9.8 % (aducanumab), 1.7 % (lecanemab), 11.8 % (donanemab) when employing thin-slice SWI. This corresponds to a relative increase of 30 % compared to T2*-GRE.

**Table 1:** The ARIA-H microhemorrhage severity distribution is impacted by the choice of MRI sequence. For each severity (none, mild, moderate, severe), proportions resulting from Monte Carlo simulations of the number of detected microhemorrhages are presented for three drugs (aducanumab, lecanemab, donanemab) and three MRI sequences (T2*-GRE, thick-slice SWI (TSWI), thin-slice SWI (tSWI)). The proportion of moderate-or-severe cases is lowest for T2*-GRE and highest for tSWI.

therapy	sequence	Simulated ARIA-H Microhemorrhage Severity Distribution				
		None	mild	moderate	severe	*moderate-or-severe*
aducanumab	T2*-GRE	82.1 %	12.3 %	3.2 %	2.4 %	*5.6 %*
	TSWI	79.7 %	12.5 %	4.0 %	3.8 %	*7.8 %*
	tSWI	78.5 %	12.5 %	5.1 %	3.9 %	*8.9 %*
lecanemab	T2*-GRE	87.0 %	8.4 %	2.4 %	2.2 %	*4.6 %*
	TSWI	85.3 %	8.5 %	3.4 %	2.7 %	*6.2 %*
	tSWI	84.5 %	8.4 %	4.3 %	2.8 %	*7.0 %*
donanemab	T2*-GRE	76.1 %	15.1 %	4.5 %	4.3 %	*8.8 %*
	TSWI	73.4 %	14.9 %	6.7 %	5.0 %	*11.7 %*
	tSWI	71.6 %	14.9 %	8.0 %	5.5 %	*13.5 %*

These observations were discussed in a panel among experts (A.M.F., W.N.G, P.S., F.B, S.S., J.C.). It was noted that guidelines on management of amyloid-β–directed monoclonal antibody therapies for Alzheimer’s disease were derived solely from evaluation on the T2*-GRE sequence. Given the significant difference in information derived from T2*-GRE and SWI, the guidance provided by the clinical trials cannot be applied to protocols using only SWI sequences. Currently, only T2*-GRE allows alignment with the clinical trials and the US Food and Drug Administration (FDA) labeling of approved amyloid-β-directed antibody therapies. With increased use of SWI in radiology practices, it is vital that radiologists are aware of the non-equivalence of the two sequences when grading ARIA-H severity. Education on appropriate imaging guidelines is needed to enhance management of amyloid-β-directed antibody therapies.

Risk assessment criteria before treatment initiation also need reevaluation in view of the higher sensitivity of SWI and its potential predictive value for ARIA risk. SWI might allow a better identification of the population at lower risk for ARIA and could be seen as a valuable addition to T2*-GRE as the baseline for post-treatment follow-up or could result in denying treatment to patients who are appropriate treatment candidates.

## CRediT authorship contribution statement

**Diana M. Sima:** Conceptualization, Formal analysis, Methodology, Writing – original draft. **Thanh Vân Phan:** Data curation, Investigation, Writing – original draft, Writing – review & editing. **Ana M. Franceschi:** Conceptualization, Writing – review & editing. **Wende N. Gibbs:** Conceptualization, Writing – review & editing. **Frederik Barkhof:** Conceptualization, Writing – review & editing. **Philip Scheltens:** Conceptualization, Writing – review & editing. **Stephen Salloway:** Conceptualization, Writing – review & editing. **Jeffrey Cummings:** Conceptualization, Writing – review & editing. **Wim Van Hecke:** Conceptualization, Supervision, Writing – review & editing. **Dirk Smeets:** Conceptualization, Investigation, Methodology, Supervision, Writing – original draft, Writing – review & editing.

## Declaration of competing interest

The authors declare the following financial interests/personal relationships which may be considered as potential competing interests:

Diana Sima reports a relationship with icometrix that includes: employment. Thanh Van Phan reports a relationship with icometrix that includes: employment. Ana M. Franceschi reports a relationship with Biogen, Eli Lilly, Eisai, Roche Genentech, icometrix, Cortechs, Life Molecular Imaging, Blue Earth Diagnostics, Siemens Healthineers, MIM Software, Cardinal Health that includes: consulting or advisory and funding grants. Frederik Barkhof reports a relationship with Biogen, Prothena, Eisai, Roche, IXICO, Combinostics, Merck that includes: consulting or advisory and funding grants. Philip Scheltens reports a relationship with EQT Lifesciences that includes: employment. Philip Scheltens reports a relationship with Vivoryon, Novo Nordisk, FujiFilm Toyama Chemical, Alzheon, Novartis Cardiology, AC Immune, UCB, ImmunoBrain Checkpoint that includes: board membership. Philip Scheltens reports a relationship with Alzheon, Brainstorm Cell, Green Valley that includes: consulting or advisory. Stephen Salloway reports a relationship with Biogen, Lilly, Roche, Eisai, Novartis, NovoNordisk that includes: funding grants. Stephen Salloway reports a relationship with Prothena, AbbVie, Acumen, CognitionRX, Kisbee that includes: consulting or advisory. Jeffrey Cummings reports a relationship with Acadia, Alkahest, AlphaCognition, AriBio, Biogen, Cassava, Cerecin, Cortexyme, Diadem, EIP Pharma, Eisai, GemVax, Genentech, Green Valley, GAP Innovations, Grifols, Janssen, Karuna, Lilly, Lundbeck, LSP, Merck, NervGen, Novo Nordisk, Oligomerix, Ono, Otsuka, PRODEO, Prothena, ReMYND, Resverlogix, Roche, Sage Therapeutics, SignantHealth, Suven, TrueBinding, Vaxxinity that includes: consulting or advisory. Wim Van Hecke reports a relationship with icometrix that includes: board membership. Dirk Smeets reports a relationship with icometrix that includes: employment. If there are other authors, they declare that they have no known competing financial interests or personal relationships that could have appeared to influence the work reported in this paper.
